# Protocol for the Development and Initial Validation of the COG-IMPACT Tool: A Purpose-Built Unmet Needs Assessment for Cancer-Related Cognitive Impairment

**DOI:** 10.3390/mps7040054

**Published:** 2024-07-10

**Authors:** Darren Haywood, Moira O’Connor, Frank D. Baughman, Alexandre Chan, Raymond J. Chan, Evan Dauer, Haryana M. Dhillon, Ashley M. Henneghan, Blake J. Lawrence, Maryam Lustberg, Janette L. Vardy, Susan L. Rossell, Nicolas H. Hart

**Affiliations:** 1 Human Performance Research Centre, INSIGHT Research Institute, Faculty of Health, University of Technology Sydney (UTS), Moore Park, NSW 2021, Australia; 2 Department of Mental Health, St Vincent’s Hospital Melbourne, Fitzroy, VIC 3065, Australia; 3 Department of Psychiatry, Faculty of Medicine, Dentistry and Health Sciences, University of Melbourne, Parkville, VIC 3010, Australia; 4 School of Population Health, Curtin University, Bentley, WA 6102, Australia; 5 School of Pharmacy and Pharmaceutical Sciences, University of California, Irvine, CA 92619, USA; 6 School of Nursing, Queensland University of Technology (QUT), Kelvin Grove, Brisbane, QLD 4059, Australia; 7 Cancer Care Services, Royal Brisbane and Women’s Hospital, Herston, QLD 4006, Australia; 8 Faculty of Science, School of Psychology, Psycho-Oncology Cooperative Research Group, The University of Sydney, Sydney, NSW 2006, Australia; 9 Centre for Medical Psychology & Evidence-Based Decision-Making, Sydney, NSW 2006, Australia; 10 School of Nursing, The University of Texas at Austin, Austin, TX 78701, USA; 11 Department of Oncology, Dell Medical School, The University of Texas at Austin, Austin, TX 78701, USA; 12 School of Medicine, Yale University, New Haven, CT 06520, USA; 13 Faculty of Medicine and Health, The University of Sydney, Sydney, NSW 2006, Australia; 14 Centre for Mental Health and Brain Sciences, Swinburne University of Technology, Hawthorn, VIC 3122, Australia; 15 Caring Futures Institute, College of Nursing and Health Sciences, Flinders University, Adelaide, SA 5042, Australia; 16 Cancer and Palliative Care Outcomes Centre, Faculty of Health, Queensland University of Technology (QUT), Brisbane, QLD 4000, Australia; 17 Exercise Medicine Research Institute, School of Medical and Health Science, Edith Cowan University, Joondalup, WA 6027, Australia; 18 Institute for Health Research, University of Notre Dame Australia, Fremantle, WA 6160, Australia

**Keywords:** cancer-related cognitive impairment, cancer fog, qualitative, mixed methods, measure development, health professionals, cancer survivors, quality of life, unmet needs, challenges

## Abstract

(1) *Background*: A significant proportion of cancer survivors report experiencing a cognitive ‘fog’ that affects their ability to think coherently and quickly, and reason with clarity. This has been referred to as cancer-related cognitive impairment (CRCI). CRCI has extensive impacts on the daily lives of people living with or beyond cancer, including occupational, social, and psychological functioning. Oncology health professionals report feeling under-resourced to effectively assess the needs of an individual with CRCI and then provide optimal care and referral. (2) *Methods*: The objective of this project is to develop and provide an initial validation of the first purpose-built unmet needs assessment for CRCI: the Unmet Needs Assessment of Cancer-Related Cognitive Impairment Impact (COG-IMPACT). We will use a multiple-stage, co-design, mixed-methods approach to develop and provide an initial validation of the COG-IMPACT. (3) *Results*: The primary anticipated result of this research is the production of the COG-IMPACT, the first purpose-built unmet needs assessment for CRCI. The assessment could be used by health professionals to understand the unmet needs and facilitate optimal care and referral for cancer survivors, by survivors to elucidate their supportive needs and advocate for their care, and by researchers to examine the correlates of unmet needs relating to CRCI, as well as how best to support people with CRCI.

## 1. Introduction

Many cancer survivors report experiencing a cognitive ‘fog’ that affects their ability to think coherently and quickly, and reason with clarity [1,2,3]. This has been referred to as cancer-related cognitive impairment (CRCI) and has been reported in up to 75% of cancer survivors [1,2]. Evidence demonstrates that CRCI can occur across tumour types and stages as well as across cancer treatments, including chemotherapy hormone therapy, radiation therapy, immunotherapy, and surgery [4,5]. Even cancer survivors who are treatment naïve (i.e., yet to receive any cancer treatment) can experience CRCI [4,5]. A body of research suggests that CRCI may be the consequence of cancer itself, cancer treatments, and the psychological impacts of cancer and its treatments, such as distress [4,6,7]. Relatedly, mental health challenges are consistently linked with cognitive dysfunction across populations [8,9,10,11,12], and cancer survivors are at a greater risk of experiencing mental ill-health [13,14,15,16,17]. Therefore, it has been suggested that mental health challenges and CRCI may have a bidirectional functional relationship, each contributing toward the development and maintenance of the other [4,18]. Irrespective of the aetiology of CRCI, its effects on the overall well-being of individuals with cancer appear to affect multiple aspects of their lives [19,20,21,22]. This can be illustrated by two quotes from Henderson et al. [19], the first from a person 1 year post-chemotherapy, and the second from a person 6.5 years post-chemotherapy: (1) “He used to really adore me … but now he thinks that I’m very … I’m a bit slow.” [19] and (2) “I think there is a piece that’s lost … it’s like a bereavement in a sense. Part of me is lost or dormant” (p. 4). Overall, the evidence suggests that CRCI has extensive impacts on the daily lives of people living with or beyond cancer, including occupational, social, and psychological functioning [20], and this has resulted in cognitive functioning being included as a rehabilitation target in the World Health Organisation’s Package of Interventions for Rehabilitation (Cancer) [23].

A variety of treatments and supportive care approaches have been developed to minimise the severity of CRCI through treatments, or minimise the impact of CRCI through addressing the needs of cancer survivors. These include cognitive behavioural therapy, mind–body interventions, cognitive remediation training, physical activity and exercise, as well as pharmacological treatment, support groups, and family/carer supportive care [2,3,4,5,24,25,26,27]. There are also a range of tools designed to assess the presence and/or severity of CRCI, including both objective neurocognitive testing, and subjective self-report measures, with some of these including score ‘thresholds’ to facilitate the determination of CRCI (see [4,24,28]). However, currently, no specific assessment tool exists to determine the unmet supportive and informational needs of someone facing the challenges specifically associated with CRCI [20]. Health professionals show awareness of CRCI and its impacts; however, they also report feeling under-resourced to effectively assess the unmet needs of an individual with CRCI and then provide optimal care and referral [20,29]. The existing unmet needs assessment tools for cancer survivors are typically broad in scope and encompass domains that may be inappropriate or unnecessary for this purpose (i.e., sexual functioning, numbness or tingling), as well as not providing information specific to the impacts of CRCI [30,31]. Conversely, there are a small number of available single-domain assessments that examine cognitive impacts for cancer survivors on narrow domains such as work [32]. These issues of excessive breadth or specificity may deter health professionals from utilising them for the assessment of people with CRCI [29]. Optimal care for CRCI may be highly individualised and dependent on their specific needs and context [4]. To ensure the delivery of optimal care, oncology health professionals require a purpose-built unmet needs assessment for CRCI that will foster and facilitate discussions, guide assessments, and facilitate the choice of appropriate referral pathways and support services. 

The objective of this project is to develop and provide an initial validation of the first purpose-built unmet needs assessment for CRCI: the Unmet Needs Assessment of Cancer-Related Cognitive Impairment Impact (COG-IMPACT).

## 2. Design

This project takes a mixed-methods design informed by the approaches taken to develop established oncology unmet needs assessment tools (e.g., [30]). However, this measurement development project incorporates additional steps and approaches, as well as a greater emphasis on co-design, involving cancer survivors and oncology health professionals. Qualitative methods used include interviews and open-text responses, while quantitative methods involve the use of surveys incorporating validated measures. This is important to ensure the needs assessment is sensitive, comprehensive, relevant, and practical to the cancer survivor, and the health professional.

## 3. Procedure

**Figure 1:**
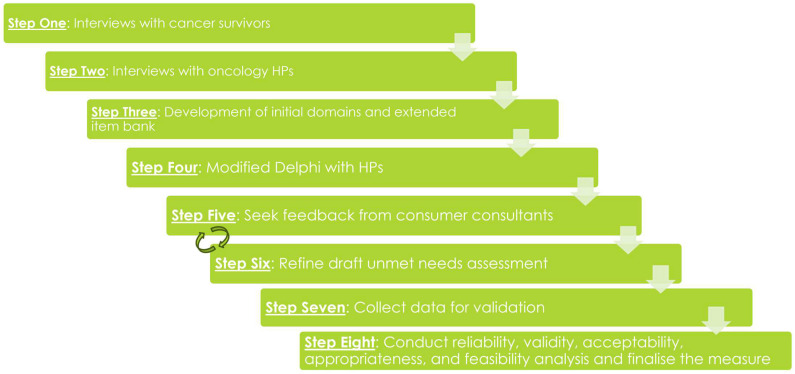
Measurement Design Process. HP = Health Professionals.

The procedure of this project follows an 8-step bottom-up design fundamentally built upon the lived experience of cancer survivors and oncology health professionals. Figure 1 provides a graphical depiction of this process and we detail each step below.

### 3.1. Step #1

Semi-structured interviews with cancer survivors who have indicated experiencing CRCI symptoms. Interviews explored specific impacts CRCI may have had on their lives, their unmet needs, supports that were or are being provided, supports they desired or still desire, and their perceptions of the assessment of their needs and how they believe this may have been improved. 

### 3.2. Step #2

Interviews with a diverse range of health professionals who work with people affected by cancer, including people following curative-intent treatment, who experience CRCI. The interviews discussed health professionals’ experiences and perceptions of CRCI and the impact it had on their patients as well as current approaches, barriers, decision-making processes, limitations on assessment and support provision, factors that would help their assessment, and decision making regarding service provision and referral for people experiencing CRCI. Health professional perspectives are fundamental in obtaining a nuanced understanding of health domains including CRCI [21]. Health professionals were also asked about their perceived needs and utility of the unmet needs assessment as well as their preferences regarding the length, format, and type of the unmet needs assessment.

Steps #1 and #2 were completed by the time of this submission and their results were published on 8 November 2023, see [20].

### 3.3. Step #3

Development of initial domains and extended item bank of the needs assessment informed by the interviews with cancer survivors and health professionals, as well as the existing literature. 

### 3.4. Step #4

Seek feedback from oncology health professionals and experts on the developed items through a modified single-round Delphi method [33]. Specifically, oncology health professionals and experts will be recruited from oncology organisations, societies and groups and they will be asked to endorse their five preferred items, amongst a large bank of items, for each of the 11 sub-domains (encompassing 6 domains) identified in step #3 from the qualitative data collection in steps #1 and #2. Five items per sub-domain was chosen as it was found in the previous qualitative steps (steps #1 and #2) that this reflected health professionals’ and cancer survivors’ preferred length of the unmet needs assessment to balance comprehensiveness with practicality. The five most endorsed items for each sub-domain will be included in the draft unmet needs assessment. If any endorsement ties were found, the selection of the included item(s) will be based on clinical utility and content validity as judged by the investigators. Five items per sub-domain will be chosen to reflect the preferred length of the unmet needs assessment established from the interviews in steps #1 and #2.

### 3.5. Step #5

Seek feedback from cancer survivor consultants comprising people with lived experience of CRCI. Participants in step #1 of the project will be invited to provide qualitative feedback on the draft unmet needs assessment. We will use multiple rounds of text-based asynchronous Cognitive Interviewing [34]. Initially, we will utilise The Reparative Approach to Cognitive Interviewing, focusing on improving items and instructions [34]. We will then use The Descriptive Approach to Cognitive Interviewing focusing on exploring participants’ interpretations and ensuring the items measure the intended domain [34].

### 3.6. Step #6

Refine the draft unmet needs assessment based on the feedback received from the Cognitive Interviewing process in step #5. After refinement, we will repeat #5. Steps #5 and #6 will be iteratively repeated until the finalisation of the draft unmet needs assessment.

### 3.7. Step #7

Administer the unmet needs assessment to a cohort of cancer survivors who personally perceive to experience CRCI. This data collection will include clinical and demographic items, the COG-IMPACT, as well as other established measures of psychosocial well-being, occupational and social functioning, quality of life, and subjective cognitive functioning for validation purposes. The participants will be invited to re-complete the COG-IMPACT two weeks after their initial completion to assess test–retest reliability.

### 3.8. Step #8

For the initial validation of the COG-IMPACT we will conduct reliability, validity, acceptability, appropriateness, and feasibility assessment. This step will include the assessment of the measure’s factor structure, internal consistency, convergent validity, reported acceptability, appropriateness, and feasibility, and test–retest reliability.

## 4. Participants 

### 4.1. Participant Descriptions

Cancer survivor participants across all steps will be (inclusion criteria); cancer survivors who are 18 years or older, have no current evidence of disease (any cancer type), have completed all treatment for cancer with curative intent, and personally perceive to experience CRCI. Participants may be from any tumour stream and have undergone any cancer treatment. The only exclusion criterion will be the diagnosis of another neurocognitive or neurological disorder.

Oncology health professional participants will be (inclusion criteria); those who are 18 years or older and work/worked directly with cancer survivors who may experience CRCI and may include oncologists (medical, radiation, surgical), haematologists, nurses, psychiatrists, psychologists, and general practitioners, among other professions [20]. The only exclusion criterion will be health professionals who have not worked with cancer survivors. 

Participants in the interviews will be invited to be cancer survivor and health professional consultants, respectively, for steps #4 and #5 of this study. 

All participants will be required to be fluent in reading and speaking the English language and be at least 18 years of age at the time of data collection. There were no geographical restrictions on participants. 

### 4.2. Recruitment 

Participants involved in steps #1, #2, #4 and #5 (cancer survivors and health professionals) will be recruited via convenience and snowball sampling from the wider community, and via oncology organisations, research groups and societies. Convenience sampling is a type of non-probability sampling in which people are sampled because they are “convenient” sources of data for researchers. Snowball sampling is defined as a non-probability sampling technique in which the samples have traits that are rare to find. A flyer will be used to recruit cancer survivors and health professionals from the general community using social media posts. For example, the cancer survivor recruitment flyer will be posted in cancer survivorship Facebook groups. Similarly, the health professional recruitment flyer will be posted in oncology health professional Facebook groups. Additionally, oncology health professionals who will take part in the modified Delphi process in step #4 will be invited to participate via email through oncology organisations, research groups and societies. Oncology health professionals at St. Vincent’s Hospital Melbourne, who work with cancer survivors, will receive an email and/or flyer inviting them to take part in a focus group or interview if they satisfied the inclusion criteria. 

Participants involved in step #7 (i.e., cancer survivors reporting to experience CRCI) will be recruited to complete the survey online via the Prolific crowd-sourcing platform [35], and through the community (e.g., social media postings, consumer groups). Prolific is a highly respected participant sourcing platform where people create an account on Prolific and can view studies for which they may fit the inclusion criteria. They will be able to view a simple description of this study, including inclusion and exclusion criteria. If they are interested in participating, they will be provided with the link to this study, which will first provide the participant information and consent form. Participants will receive a reimbursement that is detailed in the study description prior to starting this study. Prolific has been shown to be valid and reliable and has been used for thousands of studies worldwide [36]. Participants recruited through Prolific will be reimbursed the Prolific recommended amount based on the anticipated completion time for their participation. We anticipate the survey will take approximately 15–35 min to complete. To recruit people from the general community, a flyer will be posted on social media platforms, such as cancer survivor Facebook groups. The flyer will contain a link to this study, including the participant information and consent form. In addition, consumer groups, cancer survivorship advocacy groups, and society groups will be contacted to aid recruitment. In addition, participants involved in step #7 will be invited to re-take the survey two weeks after the completion of the initial survey to assess the test–retest reliability and final validation of the developed needs assessment tool. 

### 4.3. Sample Sizes

We conducted 51 interviews across steps #1 and #2 (32 cancer survivors and 19 health professionals) [20]. We aim to recruit 20–30 oncology health professionals and experts to participate in the modified Delphi process in step #4. Guided by previous needs assessment development [37], we anticipate approximately 200–300 people who report experiencing CRCI will be recruited to complete the needs assessment measurement tool and complete additional measures to assess reliability, validity, acceptability, appropriateness, and feasibility.

### 4.4. Data Collection Methods and Measures

Semi-structured interviews with cancer survivors and health professionals were used. The interviews took place online through Teams or Zoom. Semi-structured interview guides, comprising open-ended questions were used to ensure the interviews and focus group were comprehensive while acknowledging participants would have unique insights [38,39]. Elaboration and clarification prompts were used to gain further information and examples of experiences [38,39]. The interviews were conducted by a member of the investigator team and audio recorded. Audio from the interviews and focus group were transcribed verbatim. After consent was obtained participants were asked to complete a brief demographic questionnaire to obtain background information to describe the sample. This qualitative approach is commonly used within oncology research [20,29,40].

An online survey will be used to assess the validity, reliability, and acceptability of the CRCI needs assessment. The survey will be self-report and include the following measures and sections: (1) Demographic questions (i.e., age, gender, country of residence, occupation, type of cancer diagnosis, year of diagnosis, treatment received, timeline of treatment received), experiences of cancer-related cognitive impairment (tick boxes of potential psychosocial and cognitive difficulties), and timeframe of the cognitive difficulties experienced. (2) Psychosocial, quality of life, cognitive and unmet needs measures: the Depression Anxiety, and Stress Scale (DASS-21) [41], Cancer Survivors Unmet Needs Scale (CaSUN) [42], Assessment of Quality of Life Scale [43], the Cognitive Symptom Checklist-Work (CSC-W) [32], and the PROMIS (National Institutes of Health’s Patient-Reported Outcomes Measurement Information System) Cognitive Function Scale (PROMIS-COG) [44]. The PROMIS-COG was chosen in accordance with the recommendations of the Cancer Neuroscience Initiative Working Group [28] over alternative options due to its demonstrated reliability, validity and acceptability, whilst being brief, minimising participant burden. We will also use modified versions of the Acceptability of Intervention Measure (AIM), Intervention Appropriateness Measure (IAM), and Feasibility of Intervention Measure (FIM), and administer our developed unmet needs assessment for CRCI. 

The survey will be presented in Qualtrics. Each measure has been extensively validated and used within this target population.

### 4.5. Analyses

Qualitative data were analysed using reflexive thematic analysis aided by the Nvivo software (version 14). Guidelines outlined by Braun and Clarke [38,39], will be followed. Interview transcriptions were read multiple times to ensure familiarity with the data [38,39]. Initial coding of the data to identify salient concepts was systematically conducted to organise the data into manageable sections. Potential themes were identified, and codes were collated within these [38,39]. Themes were then reviewed and refined to produce a thematic map whereby meaning and explanations were be derived from the data. Themes were then used to inform the factors of the COG-IMPACT. Themes and sub-themes relating to the assessment process and practical considerations were used to inform the structure, form and delivery guidelines of the measure. Rigor and Quality within data collection and analysis were ensured by adherence to the recommendations for qualitative research [38,39,45,46,47]. These recommendations include credibility, dependability, confirmability, and transferability.

Guided by the development and validation of previous unmet needs assessments e.g., [30]), quantitative data will be analysed using statistics software, such as SPSS, and R, and include descriptive analyses (e.g., mean age, gender count, occupation count, mean acceptability, appropriateness, and feasibility), factor analysis (e.g., principle component analysis), validity analyses (e.g., correlation analyses with other established measures), and reliability analyses (e.g., internal consistency, test–retest analysis through correlations between initial and subsequent completion of the COG-IMPACT).

## 5. Expected Results

The primary anticipated result of this research is the production of an Unmet Needs Assessment of Cancer-Related Cognitive Impairment Impact (COG-IMPACT), the first purpose-built unmet needs assessment for CRCI. The COG-IMPACT could be used by health professionals to understand the unmet needs and facilitate optimal care and referral for cancer survivors, by cancer survivors to elucidate their supportive needs and advocate for their care, and by researchers to examine the correlates of unmet needs relating to CRCI, as well as how best to support people with CRCI. We expect the final structure of the COG-IMPACT to resemble the themes derived from the qualitative components of this project. We also expect the final unmet needs assessment to demonstrate good-to-excellent reliability, validity, acceptability, appropriateness, and feasibility. The findings from this research will be published in a peer-reviewed journal and presented at academic conferences.

## 6. Future Research

Once validated within the initial target population, using related methods, we will seek to validate the COG-IMPACT in other populations including those currently undergoing treatment for curative intent, and advanced and metastatic cancer survivors using a subset of the study steps. We will also aim to develop and validate a short-form version of the COG-IMPACT, as well as develop and validate other language versions. Further, we will aim to develop an adolescent and young adult (AYA) version of the COG-IMPACT informed by the process outlined in this paper. Finally, we will aim to develop referral pathways to facilitate clear and evidence-based care informed by data collected from the COG-IMPACT. 

## Data Availability

De-identified data may be made available by reasonable request of the corresponding author and to the satisfaction of the Human Research Ethics Committee approval.
